# Divergence between Neuronal and Oligodendroglial Cell Fate, in Postnatal Brain Neural Stem Cells, Leads to Divergent Properties in Polymorphic In Vitro Assays

**DOI:** 10.3390/cells11111743

**Published:** 2022-05-25

**Authors:** Maria Anesti, Stavroula Magkafa, Efstathia Prantikou, Ilias Kazanis

**Affiliations:** Laboratory of Developmental Biology, Department of Biology, University of Patras, 26504 Patras, Greece; anestimaria@gmail.com (M.A.); stavroulamagafa@gmail.com (S.M.); theniapr@gmail.com (E.P.)

**Keywords:** neural stem cells, oligodendrocyte progenitor cells, neurogenesis, oligodendrogenesis, cell fate, microenvironment, extracellular matrix, β1-integrin, microneurotrophin bnn-20, polymorphic assays

## Abstract

Two main stem cell pools exist in the postnatal mammalian brain that, although they share some “stemness” properties, also exhibit significant differences. Multipotent neural stem cells survive within specialized microenvironments, called niches, and they are vulnerable to ageing. Oligodendroglial lineage-restricted progenitor cells are widely distributed in the brain parenchyma and are more resistant to the effects of ageing. Here, we create polymorphic neural stem cell cultures and allow cells to progress towards the neuronal and the oligodendroglial lineage. We show that the divergence of cell fate is accompanied by a divergence in the properties of progenitors, which reflects their adaptation to life in the niche or the parenchyma. Neurogenesis shows significant spatial restrictions and a dependence on laminin, a major niche component, while oligodendrogenesis shows none of these constraints. Furthermore, the blocking of integrin-β1 leads to opposing effects, reducing neurogenesis and enhancing oligodendrogenesis. Therefore, polymorphic neural stem cell assays can be used to investigate the divergence of postnatal brain stem cells and also to predict the in vivo effects of potential therapeutic molecules targeting stem and progenitor cells, as we do for the microneurotrophin BNN-20.

## 1. Introduction

In the postnatal mammalian brain, mitotically active neural stem cells (NSCs) and their progeny reside in anatomically discrete microenvironments, called stem cell niches, one of which is the subependymal zone of the lateral walls of the lateral ventricles (SEZ, also known as the ventricular-subventricular zone) [1,2]. In the SEZ of the mouse and rat brain, these NSCs give rise to committed progenitors of neuronal (neuroblasts) or oligodendroglial (oligodendroblasts) fate via transit-amplifying precursor cells. Neuroblasts enter the rostral migratory stream and migrate towards the olfactory bulbs, where they differentiate mainly into interneurons and integrate into the existing circuitry [3,4], while oligodendroblasts migrate to the adjacent corpus callosum where they progress along the oligodendroglial lineage [5,6]. The pool of NSCs in the SEZ declines over time, both in the rodent and the human brain [7,8,9,10], with the oligodendroglial output showing signs of higher resistance to ageing [6,11,12]. Although the SEZ is not anatomically separated by the adjacent tissue, it is a specialized microenvironment characterized by a specific 3-dimensional (3D) architecture of ependymal cells, NSCs and downstream progenitors, as well as blood vessels [13,14]. Also specialized is the SEZ’s composition of the extracellular matrix (ECM) [15,16,17], with one of its major components being laminin, a heterotrimeric protein, composed of α, β, and γ chains that form a cross-shape [18]. Notably, neural stem and progenitor cells produce their own ECM, including laminins, which regulate their survival and proliferation [16,19]. Integrins are major ECM receptors; they are transmembrane αβ heterodimers and have several functions in the nervous system [20]. Integrins containing a β1 subunit have been shown to be of crucial importance in the regulation of the proliferation of neural stem and progenitor cells, as well as of the structural organization of the adult niche [13,16].

In contrast to neural stem cell niches that provide a unique microenvironment supportive of multipotency, the mammalian brain parenchyma is mainly considered gliogenic [21,22]. Oligodendrocyte progenitor cells (OPCs), are broadly dispersed in the grey and white matter, exhibiting strong self-renewal capacity and supporting oligodendrocyte turnover and remyelination throughout the life-span of the rodent and human organisms [6,11,12]. They do not form groups, but rather dynamically maintain a pattern of distribution [23], although an oligovascular niche has been described [24,25] and the stiffness of their microenvironment can regulate their behavior [26]. Based on the above, we hypothesized that the divergence of cell fate (neuronal versus oligodendroglial) should be accompanied by a divergence of properties, reflecting the differential adaptation to life in the niche versus the parenchyma. The elucidation of such a correlation could offer valuable first insight on the mechanisms that control different aspects of “stemness” in the brain’s progenitor populations.

NSCs can be isolated from the SEZ and grown in vitro as 3D aggregates, called neurospheres [27,28,29]. NSCs are typically expanded and passaged as neurospheres and then plated as monolayers [30] in order to investigate their properties (for example proliferation and differentiation) [31], or for drug screening [32]. Here, we allow neurospheres to generate polymorphic cultures and we take advantage of the appearance of 3D and 2D domains in order to correlate, for the first time, the behavior of progenitors of neurogenic versus oligodendrogenic cell fate with the architecture of their microenvironment. We expand our analysis by manipulating the ECM composition of the microenvironment by adding laminin, or by blocking the function of integrin-β1. Finally, we show that this type of NSC polymorphic assay can be used in order to predict the mode of action of compounds destined to target progenitors in vivo. We test that by comparing the in vitro effects of the microneurotrophin BNN-20 with in vivo data previously generated by our group after the administration of BNN-20 in healthy and parkinsonic mice [33].

## 2. Materials and Methods

### 2.1. Animals

Adult male and female B6CBAC wild-type mice (1–3-month-old) were used for the isolation of postnatal brain neural stem cells. Mice were kept in the animal facilities of the University of Patras, in standard laboratory polyacrylic cages (three to five mice/cage), under relative humidity of 50–60%, controlled temperature (22 ± 1 °C), and a steady light–dark cycle (12 h/12 h), with free access to water and food. Their breeding and maintenance were in accordance to the European Communities Council Directive Guidelines (86/609/EEC) for the care and use of Laboratory animals as implemented in Greece by the Presidential Decree 56/2013 and approved and scrutinized by the Prefectural Animal Care and Use Committee (No: EL 13BIO04) and the Animal Welfare and Ethical Review Committee of the University of Patras.

### 2.2. Reagents, Substrates and Functional Antibodies for Cell Cultures

Gibco Dulbecco’s modified Eagle’s medium − high glucose + pyruvate (DMEM), as well as B27 and N2 culture supplements were obtained from Thermo Fisher Scientific (11995-065, 17504-044 and 17502-048, respectively). Fibroblast growth factor-2 (FGF-2) and Epidermal growth factor (EGF) were purchased from Peprotech (100-18B and 315-09). Accutase was obtained from PAN-Biotech (P10-21100). Regarding the substrates, Poly-D-Lysine (PDL) and Laminin were purchased from Thermo Fisher Scientific (A3890401) and Sigma (L2020), respectively. The integrin β1-blocking antibody (Purified Rat Anti-Human CD29) was obtained from BD Pharmingen (552828) and the IgG isotype control (553951) was also purchased from BD Pharmingen.

### 2.3. NSC Cultures

To obtain adult neural stem and progenitor cells in vitro, the SEZs of adult mice were dissected under a stereoscope, dissociated with accutase (37 °C, 20 min) and were resuspended in standard NSC proliferation medium containing high glucose DMEM medium, supplemented with 2% B27, 1% N2, FGF-2 (final concentration 20 ng/mL) and EGF (final concentration 20 ng/mL). In the presence of growth factors NSCs can be grown in the form of 3D, freely-floating aggregates with self-renewing capacity, called neurospheres and are passaged every 5–7 days.

### 2.4. Coating of Coverslips

Sterile glass coverslips were coated with PDL, or PDL+ Laminin. For PDL coating, coverslips were incubated with PDL (100 μg/mL), overnight, at 37 °C and the next day they were rinsed with sterile water and left to dry. For laminin coating, PDL-coated coverslips were incubated with laminin (50 μg/mL) for 2 h at 37 °C and they were rinsed with sterile PBS before plating neurospheres.

### 2.5. Polymorphic Neurosphere Assay

Neurospheres at three to four days post-passage (6th-7th passage) were centrifuged (600 g, 5 min), resuspended in the standard proliferation NSC medium and were seeded on coverslips (either in 24-well or 48-well plates). The next day, the medium was changed (differentiation medium: DMEM, 2% B27, 1% N2), and cells were kept for seven days and then were fixed. To control for cell numbers, respective samples were dissociated into single cells with accutase and quantified. Typically, we plated neurosphere samples with approximately 60,000 cells per well in a 48-well plate (coverslips of 10 mm diameter).

For the comparison between 2D microenvironments of the polymorphic neurosphere assay and of adherent monolayer NSC cultures, neurospheres of each sample were centrifuged, resuspended in the standard proliferation NSC medium, and were split in two. Half were plated on PDL-coated coverslips and the remainder was centrifuged, dissociated (with accutase), resuspended and plated as dissociated cells. The next day, media were changed and cells were cultured in differentiation conditions for seven days.

### 2.6. Laminin, Integrin-β1 Blocking and BNN-20 Experiments; Time-Lapse Imaging

Neurospheres were plated on PDL or “PDL + Laminin-coated” coverslips in the standard NSC proliferation medium in order to attach and migrate for 24 h and subsequently they were cultured in differentiation conditions for seven days. An integrin β1-blocking antibody (10 μg/mL) [34] was added to the differentiation medium for the first 4 days. For the BNN-20 experiment, the microneurotrophin was added to the differentiation medium (100 nM) for the whole seven days as previously described [33]. A time-lapse recording was performed using a CytoSMART device, with neurospheres seeded on glass coverslips coated with PDL (Control group) or PDL + Laminin (Laminin group). Recording began once cells were put in a differentiation medium, with an overall duration of 72 h, and images were taken every 60 min.

### 2.7. Immunocytochemistry and Antibodies

Cells were fixed with 2% paraformaldehyde (PFA) (15 min at room temperature/RT) and were processed for immunofluorescence staining using standard protocols. Briefly, cells were incubated with blocking buffer (3% BSA, 0.1% Triton x-100 from Sigma, in PBS) for 1 h at RT and primary antibody incubation (in blocking buffer) was performed for one (or two only for CC1 and PDGFRa) night at 4 °C. The next day, samples were incubated with the appropriate secondary antibodies for 2 h at RT and were mounted with mowiol. The following antibodies were used (with dilution, provider and catalogue number): rabbit anti-OLIG2 (1/300, Millipore, AΒ9610), rat anti-PDGFRa (1/100, Millipore, CBL1366) and mouse anti-CC1 (1/100, Millipore, OP80) were used to identify oligodendroglial lineage cells, OPCs and more mature oligodendrocytes, respectively. To assess neuroblasts and immature neurons, rabbit anti-DCX (1/800, Abcam, ab18723) and mouse anti-tubulin βIII (1/500, Abcam, ab7751) were used respectively. Stem and progenitor cells were identified, using a goat anti-SOX2 antibody (1/200, Santa Cruz, sc-17320) and a chicken anti-nestin antibody (1/200, Abam, 130417). To mark proliferating cells, we used a mouse anti-PCNA (1/500, Abcam, Ab29) and a rabbit anti-Ki67 antibody (1/500, Abcam, ab16667). Appropriate secondary antibodies conjugated, with fluorescence dyes, were purchased from Thermo Fisher Scientific (Molecular probes) and Biotium, raised in donkey or goat IgGs with fluorophores of 568 nm, 488 nm or 647 nm.

### 2.8. Imaging, Cell counts and Statistical Analysis

Images were taken using a Leica SP8 confocal microscope. The distinction between 2D and 3D microenvironments was performed based on DAPI, nuclear staining, by evaluating the number of stacks and the size of z-axis in different, randomly selected, optical fields, using the x63 objective lens. When cell nuclei were detected at multiple layers, at a width higher than 15 μm (≥3 steps of 5μm), the field was categorized as 3D structure; when cell nuclei were spanning at maximum 2 steps of 5 μm, the area was categorized as 2D. When working with NSC monolayers, after plating single-cell suspensions, the 2D areas were predominant. For cell counts, at least 15 random optical fields per coverslip (10 mm diameter) were acquired with the x63 objective lens. All cells immunopositive for cell type specific markers were counted and percentages were calculated. It should be noted that in differentiation conditions a high volume of apoptotic cell death is observed. The nuclei of apoptotic cells become fragmented and very densely stained with DAPI; these were not counted. For the Pearson correlation, between the numbers of Dcx+ or Olig2+ cells and the total cell number, all optical fields (per biological sample and experiment) were binned according to the number of cells (e.g., one, two, three, four Dcx + /Olig2+ cells) and the average number of total nuclei, per bin, was calculated. For cell morphology analysis, all Dcx+ cells for which the whole cell body was clearly visible in the z-stacks were identified and included. The number of processes (neurites) emerging from each cell body were manually counted. In neurites with clearly visible processes, the length of each neurite was manually measured using the respective tool of LasX (Leica) software and the total length per cell was calculated. For the oligodendroblast aggregation analysis, one Olig2+ cell was randomly chosen in each optical field and a circle of 30μm radius was drawn around the center of the nucleus. The numbers of all DAPI stained nuclei, of Olig2+ and of Olig2 + PCNA+ nuclei positioned within the circle were counted.

Statistical analyses were performed using the IBM SPSS statistical software and Microsoft Office Excel, and the graphs were constructed in the GraphPad Prism 5.0 software. Whenever possible the same cell sample was seeded in multiple coverslips and cells were grown at the same time in different conditions (control, +laminin, +integrin-β1 blocking Ab). For statistical comparisons in which all paired conditions were assessed for all biological samples (“*n*” numbers), as described above, the analyses were performed using paired Student *t*-tests (for two groups), or repeated measures ANOVA. If some samples included in the analyses did not conform to this rule (e.g., a biological sample was included only in the control group), then we used an unpaired *t*-test or one-way ANOVA, followed by post hoc tests. For the assessment of the correlation between neurogenesis or oligodendrogenesis with cell density, a Pearson correlation analysis was performed with SPSS. Data are presented as mean ± S.E.M. Probability values lower than *p* = 0.05 were considered statistically significant.

## 3. Results

### 3.1. Characterization of NSC Polymorphic Assays

**Figure 1:**
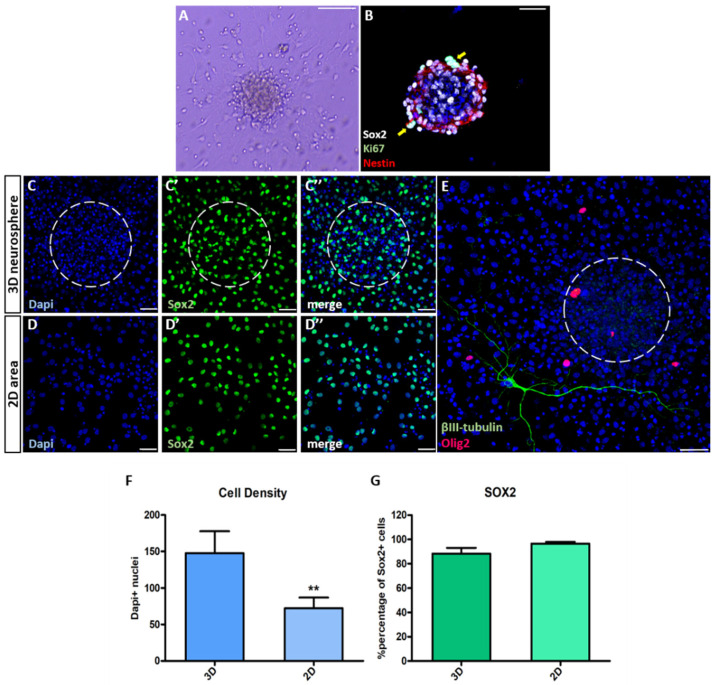
A polymorphic neurosphere assay of postnatal NSCs with a range of cytoarchitectures. (**A**) Representative brightfield image of a neurosphere in differentiation conditions. Note the cells having migrated out of the sphere, creating a 2D, monolayer, microenvironment. (**B**) Image of a neurosphere immunostained for Sox2, Nestin (neural stem and progenitor cell markers) and Ki67 (proliferation marker). The arrows indicate proliferating stem and progenitor cells (Nestin + Sox2 + Ki67+). (**C**,**D**) Representative images of 3D (**C**) and 2D (**D**) microenvironments of the polymorphic NSC assays after 7d in differentiation conditions immunostained for Sox2. (**E**) Representative image of an immature neuron (βIII+) at the periphery of a neurosphere and of oligodendrocyte lineage cells (Olig2+) inside and outside of the neurosphere. Note the absence of co-expression of markers of the two lineages. (**F**,**G**) Graphs showing total cell density (**F**) and the percentage of Sox2+ cells (in **G**), comparing between 3D and 2D microenvironments. [** *p* < 0.01, using paired *t*-test, *n* = 5–7. Error bars: SEM. Scale bars, (**A**): 60 μm, (**B**–**E**): 30 μm; delineated areas indicate where spheres were anchored. Also, note the high numbers of nuclear fragments, typical of apoptotic cells, observed as very dense DAPI+ structures. These were not counted as DAPI+ nuclei].

In order to investigate the possible correlation between the cytoarchitecture of the microenvironment and the behavior of progenitors of neuronal or oligodendroglial cell fate, we plated NSCs in the form of neurospheres and allowed the cells to differentiate for seven days (7d) by removing growth factors from the medium. We could visually detect domains of higher cell density in the places where neurospheres were initially anchored, in addition to large monolayered areas formed by cells having migrated away from the spheres (Figure 1A and Supplementary Movie S1), with proliferating stem and progenitor cells, constituting less than 0.5% of total cells and being still visible only within the spheres (Figure 1B). By analyzing the distribution of Dapi-stained nuclei, using confocal microscopy, we were able to confirm the existence of 3D and 2D domains (Figure 1(C,C”,D,D”),E, with the 3D domains indicated by white dotted lines), with the former being significantly denser with cells (Figure 1F, *p* = 0.003, using paired *t*-test, *n* = 7). The majority of cells, irrespective of being located in 3D or 2D areas, were Sox2+ stem/progenitor cells, at similar percentages (Figure 1G, *p* = 0.072, using paired *t*-test, *n* = 5). In these “NSC polymorphic” cultures the 2D monolayers are created gradually, through cell migration; thus, differently from the usual “dissociation and plating” protocols. Therefore, we assessed if cells located in these areas behaved similarly to those in typical, adherent monolayer cell cultures. We found that the total cell density, as well as the percentage of cells of neuronal fate (marked by the expression of Doublecortin (Dcx) and/or of βIII-tubulin (βIII)), of oligodendroglial cell fate (marked by the expression of Olig2) and of progenitor identity (marked by the expression of Sox2) were similar between the two cell culture protocols (Supplementary Figure S1A–I, *p* = 0.121 for cell density; *p* = 0.399 for Dcx+; *p* = 0.077 for βIII +; *p* = 0.766 for Olig2+; *p* = 0.069 for Sox2+; using paired *t*-test, *n* = 2). In the polymorphic cultures the majority of Dcx+ cells did not co-express βIII, which marks more mature neuroblasts (approximately 16.00% of all Dcx+ cells co-expressed βIII+) (data not shown and Supplementary Figure S1J) and we never detected any cells co-expressing neuronal and oligodendroglial markers (βIII and Olig2) (Figure 1E). In addition, the presence of more mature oligodendroglial lineage cells, co-expressing Olig2 and CC1 is extremely rare (<1%), while the co-expression of Olig2 and PDGFRα was detected in more than 50% of Olig2+ cells (data not shown and Supplementary Figure S1K,L). Therefore, we concluded that in the selected conditions we detect almost exclusively neuronal and oligodendroglial progenitors at their early stages after cell fate commitment.

### 3.2. Neurogenesis Is More Dependent on the Cytoarchitecture of the Microenvironment Than Oligodendrogenesis

**Figure 2:**
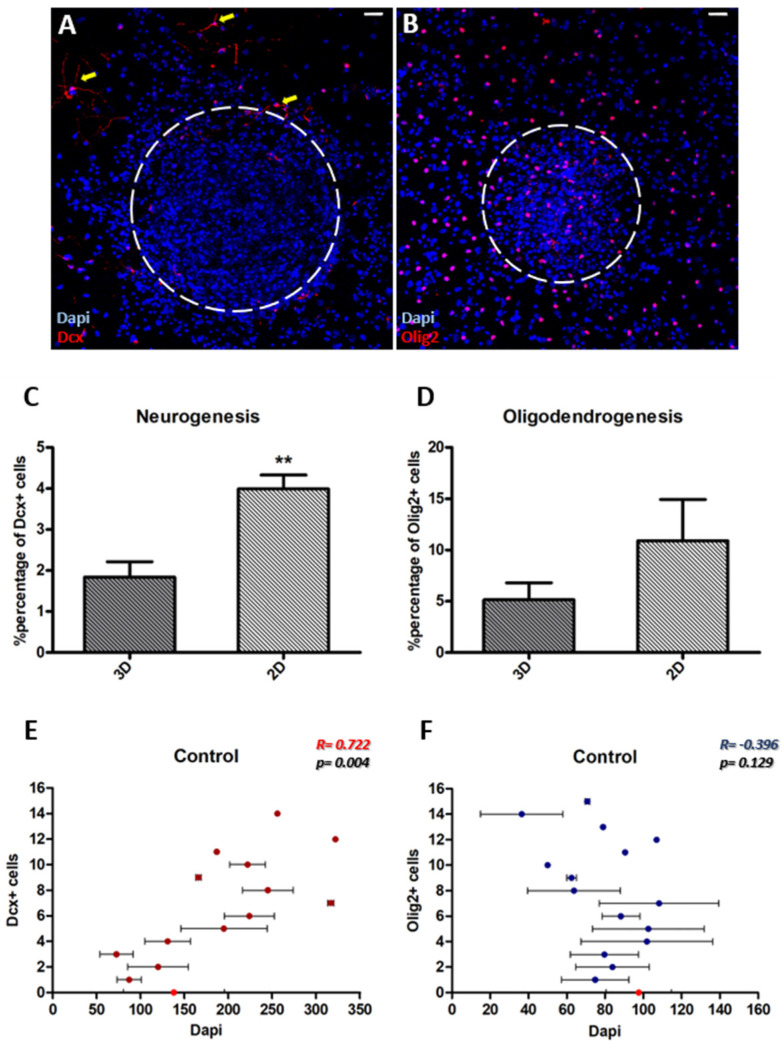
Neurogenesis is more dependent on the cytoarchitecture of the microenvironment than oligodendrogenesis. (**A**,**B**) Representative images of neurogenesis (Dcx+ cells) and oligodendrogenesis (Olig2+ cells) in polymorphic assays. Neuroblasts (indicated by yellow arrows) are detected mainly in monolayer areas but at the periphery of anchored neurospheres (**A**), while oligodendroblasts are more evenly distributed (**B**). (**C**,**D**) Graphs showing the comparison of percentages of Dcx+ and Olig2+ cells between 3D and 2D areas in polymorphic assays, with NSCs being cultured for 7d in differentiation conditions (** *p* < 0.01, using paired *t*-test, *n* = 5–6; Error bars: SEM). (**E**,**F**) Pearson correlation between the density of Dcx+ or Olig2+ cells and the total cell density, revealing a positive correlation for Dcx+ cells (*p* = 0.004, r = 0.722, *n* = 5) (**E**) and no correlation for Olig2+ cells (*p* = 0.129, r = −0.396, *n* = 6; Error bars: SEM, with light red is indicated the average cell density in optical fields with no Dcx or Olig2 positive cells) (**F**). [Scale bars: 30 μm].

In our cultures, the overall fraction of progenitors of neuronal cell fate was significantly lower than that of progenitors of oligodendroglial cell fate (2.28% ± 0.34 Dcx+ cells and 9.30% ± 2.20 Olig2+ cells, *p* = 0.033, using paired *t*-test, *n* = 5). A visual inspection of the immunostained cultures indicated that Dcx+ cells were more abundant at the periphery and away from 3D areas (Figure 2A), and our analysis confirmed that neurogenesis was significantly higher in 2D areas (Figure 2C, *p* = 0.001, using paired *t*-test, *n* = 5). In contrast, oligodendrogenesis was similar in both area types (Figure 2B,D, *p* = 0.086, using paired *t*-test, *n* = 6). In order to investigate the distribution of these cells in more detail, we evaluated the correlation between the density of Dcx+ or Olig2+ cells and the total cell density around these cells. To achieve that we performed a Pearson correlation analysis using all of the optical fields we had imaged, calculating for each biological sample and experimental repeat the average number of Dapi+ nuclei per field, binning together the fields with a specific number of Dcx+ or Olig2+ cells, starting with the fields without any such cells (shown with a red dot-point in Figure 2E,F). Our analysis revealed a positive correlation for neurogenesis (Figure 2E, r = 0.722, *p* = 0.004, Pearson correlation), indicating that numbers of Dcx+ cells were directly proportional to cell density. In contrast, no correlation was found for OPCs (Figure 2F, r = −0.396, *p*=0.129, Pearson correlation). Importantly, Olig2+ cells were appearing in overall less dense areas of the cultures (average cell density around Dcx+ and Olig2+ cells: 192 ± 20.82 and 81.1 ± 5.20, respectively; *p* < 0.001, using unpaired *t*-test). It is noted that optical fields were imaged randomly and that average cell densities in the fields void of Dcx+ and Olig2+ cells were not statistically different (light red dots on X axes, in Figure 2E,F).

In vivo analyses have shown that OPCs assume a tightly-regulated distribution in the tissue [23], an impression also gained by looking at our cell cultures that had the higher densities of Olig2+ cells (Figure 2B). In order to investigate the level of grouping of oligodendroblasts we mapped the appearance of Olig2+ cells at the immediate area (within a radius of 30μm) around random Olig2+ cells. Our analysis confirmed the absence of oligodendroblast aggregation, as an average 11.64 ± 1.32 cells were found within a 30 μm radius around an oligodendroblast, with the presence of other Olig2+ cells being extremely rare (less than 1% of all the proximally detected cells) (Supplementary Figure S2A, control bar at the left).

### 3.3. The addition of Laminin Affects the Cytoarchitecture of the Cultures

**Figure 3:**
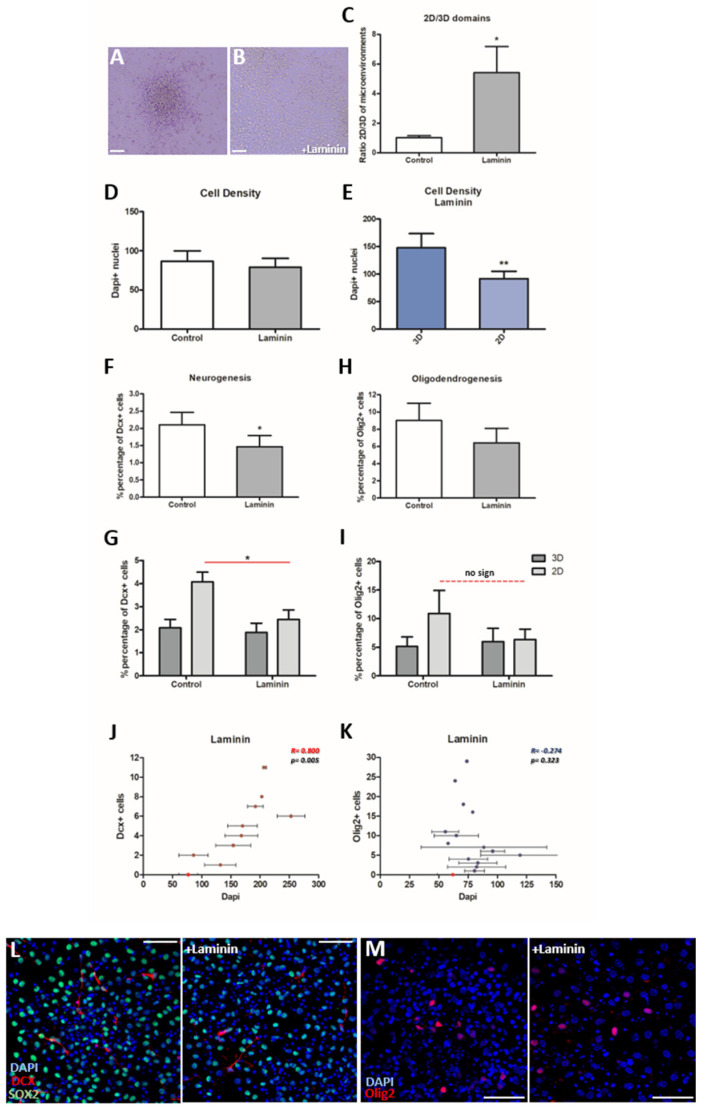
Effect of laminin on the cytoarchitecture and cell fate of NSCs in the polymorphic neurosphere assay. (**A**,**B**) Characteristic brightfield images of polymorphic NSC cultures after in vitro differentiation in control conditions (**A**) or in the presence of laminin (**B**). Note the change towards a 2D structure in (**B**). (**C**) Graph showing the ratio of 2D/3D domains in the polymorphic assay, after 7d in differentiation, comparing between the “Control” and “+Laminin” groups (* *p* < 0.05, using paired *t*-test, *n* = 7). (**D**,**E**) Graphs showing the comparison of cell density between “Control” and “+Laminin” groups (in **D**) and 3D-2D microenvironments only in the presence of laminin (in **E**) (** *p* < 0.01, using paired *t*-test, *n* = 7). (**F**,**G**) Graphs showing the percentage of Dcx+ cells in the whole culture (in **F**) and separate for the different microenvironments (**G**) (* *p* < 0.05, using paired *t*-test, *n* = 4–5). (**H**,**I**) Graphs showing the percentage of Olig2+ cells in the whole culture (in **H**) and separate for the different microenvironments (**I**) (*p* > 0.05, using paired *t*-test, *n* = 6–7). (**J**,**K**) Pearson correlation analysis for the density of Dcx+ or Olig2+ cells and the total cell density in the presence of laminin revealed a positive correlation for Dcx+ cells (*p* = 0.005, r = 0.8, *n* = 5) (in **J**) and no correlation for Olig2+ cells (*p* = 0.323, r = −0.274, *n* = 6) (in K). (**L**,**M**) Representative images of NSCs differentiated for 7d with or without the addition of laminin and after immunostaining for different markers. [Error bars: SEM; in (**J**,**K**) with light red is indicated the average cell density in optical fields with no Dcx or Olig2 positive cells; scale bars: (**A**,**B**): 20 μm, (**L**,**M**): 40 μm].

The NSC niche is a specialized microenvironment that is particularly rich in ECM molecules such as laminin [16]. In order to assess the level of dependence of neurogenesis and oligodendrogenesis to the niche microenvironment, we coated the glass coverslips with laminin. We observed a dramatic change in the cytoarchitecture of the cultures (Figure 3A,B) that became more homogeneously two-dimensional (Supplementary Movie S2), with the ratio of 2D/3D domains becoming significantly increased (Figure 3C, *p* = 0.045, using paired *t*-test, *n* = 7). The total cell density of the cultures did not change between the control and the “+laminin” groups (Figure 3D, *p* = 0.115, using paired *t*-test, *n* = 7) and the fewer remaining 3D areas were still significantly denser than the 2D areas (Figure 3E, *p* = 0.007, using paired *t*-test *n* = 7). Thus, the addition of laminin led to a redistribution of NSCs by reducing the polymorphic character of the cultures and expanding the 2D domains. 

### 3.4. Neurogenesis Is Significantly Decreased in the Presence of Laminin, While Oligodendrogenesis Is Not Affected

We assessed whether cell fate choices of adult NSCs were affected in the “+laminin” conditions and we found that neurogenesis was significantly reduced (Figure 3F, *p* = 0.023, using paired *t*-test, *n* = 5). Notably, after further analysis, this reduction was shown to be significant, specifically in the 2D areas (Figure 3G, *p* = 0.014, using paired *t*-test, *n* = 4) in which levels of neurogenesis converged towards those observed in 3D areas of the control group (Figure 3G, *p* = 0.157, using paired *t*-test, *n* = 4). Hence, the difference between 3D and 2D areas, in terms of neurogenesis, was not present any more in the “+laminin” group (Figure 3G, *p* = 0.312, *n* = 4). On the other hand, oligodendrogenesis was not affected significantly by the addition of laminin (Figure 3H, *p* = 0.078, using paired *t*-test, *n* = 7), in both 3D and 2D microenvironments (Figure 3I; *p* = 0.558, *n* = 7 and *p* = 0.133, *n* = 6, respectively). Finally, the correlation analysis revealed no changes in the presence of laminin, with a strongly positive correlation for neurogenesis (Figure 3J, r = 0.800, *p* = 0.005, Pearson correlation) and no correlation for oligodendrogenesis (Figure 3K, r = −0.274, *p* = 0.323, Pearson correlation). Similarly, the divergent preference of oligodendrogenesis for less dense areas did not change in the presence of laminin (cell density around Dcx+ and Olig2+ cells: 164 ± 17.36 and 77 ± 4.24, respectively; *p* < 0.00, using unpaired *t*-test). These results show that only neurogenesis was affected by the presence of a niche-like ECM molecule (Figure 3L,M), switching to a more “3D-like” behaviour irrespective of the dominance of the 2D architecture.

### 3.5. Neurogenesis and Oligodendrogenesis Are Differentially Affected by Blocking Integrin-Β1 Function

**Figure 4:**
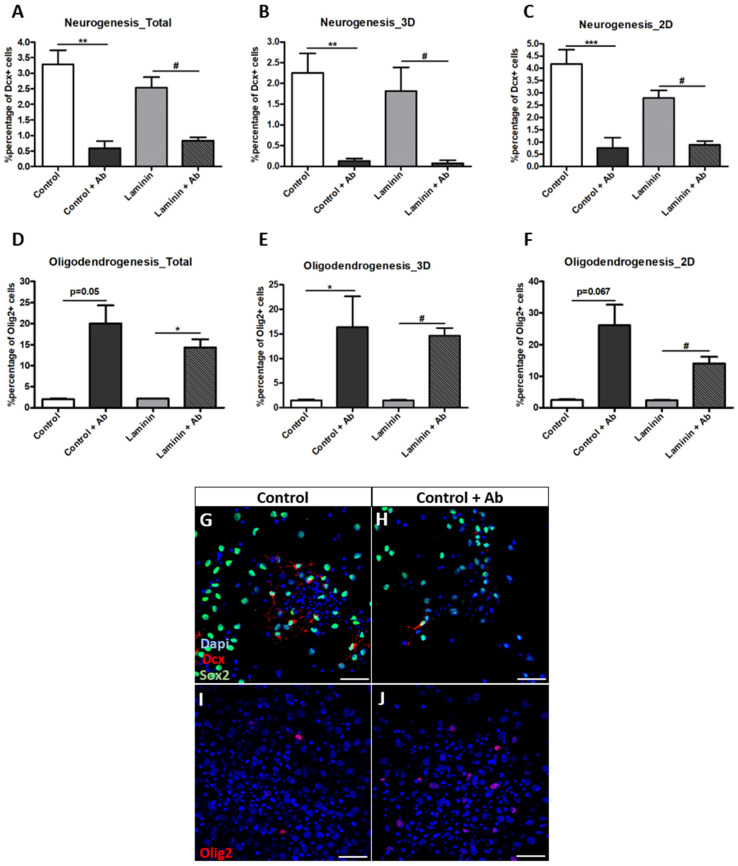
The blocking of integrin-β1 further enhances the divergence between neurogenesis and oligodendrogenesis in polymorphic assays in vitro. (**A**–**C**) Graphs showing the total percentage of Dcx+ cells in the cultures (**A**) and within separate microenvironments (**B**,**C**) (** *p* < 0.01, *** *p* < 0.001 comparison between “control” and “control + Ab” groups; # *p* < 0.05 comparison between “Laminin” and “Laminin + Ab” groups). Statistical analysis was performed by one-way ANOVA, followed by LSD post hoc tests; *n* = 3 for total/2D microenvironments and *n* = 2–3 for 3D microenvironments. (**D**–**F**) Graphs showing the total percentage of Olig2+ cells in the cultures (**D**) and within separate microenvironments (**E**,F) (* *p* < 0.05 comparison between “control” and “control + Ab” groups; # *p* < 0.05 comparison between “Laminin” and “Laminin + Ab” groups). A statistical analysis was performed by: (1) a repeated measures ANOVA for total and 2D microenvironments (*n* = 3 samples per group) and (2) one-way ANOVA followed by LSD post hoc test for 3D microenvironments (*n* = 2–3). (**G**–**J**) Representative images of NSCs differentiated for 7d with or without the addition of the integrin-β1 blocking antibody to the differentiation medium and after immunostaining for Dcx (**G**,**H**) or Olig2 (**I**,**J**). [Error bars: SEM; scale bars, 40 μm].

In order to investigate further if laminin directly plays a role on NSC fate choices, we performed cell cultures in which we blocked the function of integrin-β1, a major subunit of integrins, expressed by NSCs in the SEZ [16]. Interestingly, our analysis revealed that the blocking of integrin-β1 led to a significant reduction in neurogenesis in control conditions (“control + Ab” group; Figure 4A, *p* = 0.002, using one way ANOVA analysis, *n* = 3) and this effect was present in both 3D (*p* = 0.007, one way ANOVA, *n* = 2–3) and 2D (*p* = 0.000, one way ANOVA, *n* = 3) microenvironments (Figure 4B,C). Moreover, the inhibition of integrin-β1 in the presence of laminin (“laminin + Ab” group) also led to a significant reduction in neurogenesis (Figure 4A, *p* = 0.03, one way ANOVA, *n* = 3) in both 3D (Figure 4B, *p* = 0.026, *n* = 2–3) and 2D (Figure 4C, *p* = 0.011, *n* = 3) areas. On the other hand, oligodendrogenesis was significantly increased in the “control + Ab” group (Figure 4D, *p* = 0.053, using repeated measures ANOVA, *n* = 3), with a statistically significant effect only in the 3D areas (Figure 4E, *p* = 0.017, one way ANOVA, *n* = 2–3), as well as in the “laminin+ Ab” group (Figure 4D, *p* = 0.024, repeated measures ANOVA, *n* = 3), in both domain types of the culture (Figure 4E; 3D: *p* = 0.043, one way ANOVA, *n* = 2–3 & Figure 4F; 2D: *p* = 0.031, repeated measures ANOVA, *n* = 3), reinforcing the strong divergence of the two cell fates, here in relation to the role of integrin-β1. When the experiment was repeated using an isotype-matched antibody we observed no differences in the percentages of Dcx+ and Olig2+ cells (data not shown). Since in the “+laminin” and the “+laminin+Ab” groups neurogenesis was found to be significantly reduced, we investigated in more detail if that was also reflected in the morphology of neuroblasts. We quantified the number and the total length of neurites per Dcx+ cell in all the conditions, and we found no significant changes, even though the neurites’ length in the “control + Ab” group showed a strong trend towards being reduced (Supplementary Figure S2B–E). On the other hand, the significant increase in the numbers of Olig2+ cells in the presence of the blocking antibody prompted us to investigate the aggregation behavior of oligodendroblasts. Our analysis revealed a significant increase when compared to the “control” and “+laminin” conditions, in the appearance of Olig2+ cells within the 30μm radius around Olig2+ cells, with almost 20% of all cells around an oligodendroblast being Olig2+ (Supplementary Figure S2A). Overall, our results show that the laminin-dependent decrease of neurogenesis is not mediated by integrin-β1 and that the acquisition of neuronal and oligodendroglial cell fate by NCSs is dependent on integrin-β1, but in opposite directions.

### 3.6. BNN-20 Affects Neurogenesis and Oligodendrogenesis Only in the 2D Microenvironments

**Figure 5:**
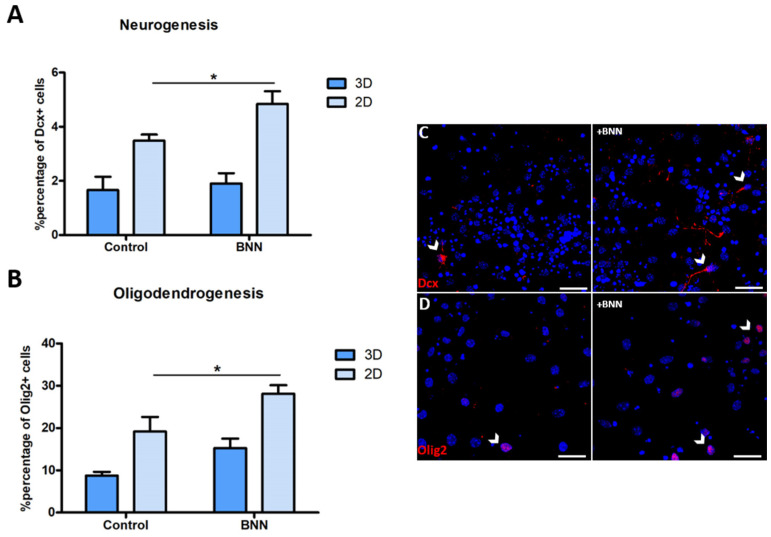
BNN-20 promotes neuronal and oligodendroglial cell fate only in the 2D microenvironments of polymorphic assays. (**A**,**B**) Graphs showing the percentage of Dcx+ (in **A**) and Olig2+ (in **B**) cells in 3D and 2D microenvironments, without or with the addition of BNN-20 to the differentiation medium for 7d (* *p* < 0.05, using paired *t*-test, *n* = 3). (**C**,**D**) Characteristic images of NSCs, differentiated without or with the addition of BNN-20, after immunostaining for Dcx (**C**) and Olig2 (**D**) (some positive cells are indicated with arrowheads). [Error bars: SEM; Scale bars, 30 μm].

We have previously shown that BNN-20, a synthetic microneurotrophin, enhances NSC differentiation in vitro, while its in vivo effects are limited to the substantia nigra parenchyma, without any effects in the SEZ and the hippocampal niches [33]. To assess if this differential activity of BNN-20 can be detected in our polymorphic assays, we differentiated NSCs, with or without the addition of the microneurotrophin, for seven days. Our results revealed that BNN-20 led to a significant increase in the percentage of Dcx+ neuroblasts, as well as of Olig2+ oligodendroblasts in the 2D areas (neurogenesis and oligodendrogenesis: *p* = 0.043, using paired *t*-test, *n* = 3) and showed no significant effects within the 3D areas (neurogenesis: *p* = 0.739; oligodendogenesis: *p* = 0.161, using paired *t*-test, *n* = 3) (Figure 5). 

## 4. Discussion

Two main populations of stem cells exist in the postnatal mammalian brain: multipotent NSCs, with neurogenesis being their main lineage output, that reside in stem cell niches and the essentially unipotent OPCs, dispersed throughout the parenchyma. The SEZ pool of NSCs declines with ageing, more dramatically in the human brain [7,8,9,10], with only its oligodendrogenic output exhibiting signs of resistance [6,11]. On the other hand, OPCs retain high self-renewing potential even in the aged brain [6]. The SEZ is a specialized microenvironment in terms of ECM and cell architecture [15,16,17], populated mainly by NSCs and their progeny, and with major ECM components also generated by the same cells [16]. Our driving hypothesis has been that the divergent properties of NSCs and OPCs are correlated to their adaptation to life in niches and in the parenchyma, respectively. If this were to be true, there must be a concurrent establishment of differential “stemness” properties and of microenvironment preferences, and the ability to manipulate the former will be reflected on the latter and vice versa. Here, we describe a polymorphic NSC culture assay and we show that neurogenesis exhibits different levels of spatial restrictions and of dependency to the niche ECM microenvironment when compared to oligodendrogenesis, with these differences being compatible to the in vivo behavior of these progenitor pools. Furthermore, we provide proof-of concept that polymorphic NSC cultures can be used as a first-line, effectiveness-predicting tool when testing NSC-targeting molecules (summarized in Table 1).

Neuronal commitment, in SEZ-derived NSCs, is accompanied by strong spatial restrictions/requirements. Progress down the neuronal pathway is marginal within progenitor-denser, “niche-like” structures, with neuroblasts appearing more robustly in 2D areas, but at the periphery of the 3D domains, and exhibiting a positive correlation with cell density. In other words, the first step in the path towards the neuronal commitment is for the neural progenitor to migrate out of the niche. The distribution of these progenitors is more likely regulated at the level of cell-to-cell contacts and local signaling [35], but once established, neurogenesis seems to progress passively, without specific spatial restrictions. These observations are in agreement with the well-described dependence of postnatal brain neurogenesis on the presence of niches [2,17,36] and on progenitor-to-progenitor interactions [37]. Notably, definite commitment to the neuronal fate (marked by the expression of Dcx) happens in neuroblasts, the daughter cells of transit amplifying progenitors, which are cells characterized by a lower degree of anchorage to SEZ cell elements and a propensity for migration out towards the parenchyma [13,38]. The role of the cytoarchitecture in the regulation of neurogenesis has also been highlighted in a recent study that reported a higher potential of human induced pluripotent stem cell lines to generate cortical neural progenitors and neurons in 3D (organoid) versus 2D (monolayer) conditions; also showing long-term differences in the specification and differentiation of neurons and revealing further a fundamental role of cell-to-cell interactions of progenitors in maintaining intercellular signaling mechanisms and transcriptional regulations that control the cellular and regional fate commitment of neuronal progenitors between the two conditions [35]. On the other hand, oligodendrogenesis exhibited a different pattern of regulation. It was detected equally in both 3D and 2D domains, but within a narrower range of cell densities. Importantly, it showed no direct proportionality to cell density and the absence of aggregation, indicating a more active spatial regulation of the establishment of oligodendroglial fate commitment. This is in accordance to in vivo observations, according to which OPCs and oligodendroblasts are detected throughout the brain (from the middle of the SEZ (as a subset of transit amplifying progenitors [39]), to the parenchyma [40]), but actively maintaining their density [23]. Our data showed that the aggregation restriction was partially reversed after the blocking of integrin-β1.

Laminin constitutes a major ECM component within the SEZ, suggesting that it is a key regulator of NSC behavior [16]. When we enriched the culture microenvironment with laminin, we created an artificial “2D niche” structure (2D in architecture, niche-like in ECM composition), and our analysis showed that the levels of neurogenesis were reduced and converged towards those observed in 3D areas. Thus, we conclude that the effects of the ECM composition of the microenvironment dominate over the effects of architectural traits and of cell-to-cell interactions. It is noteworthy that the effects of laminin as a regulator of both differentiation and proliferation of progenitor cells are quite intricate [41]. Laminin has been shown to enhance progress towards neuronal differentiation in human and mouse-derived NSCs, but that study was performed in uniform 2D cultures with neuronal output investigated at longer time points and with the readout being neuronal maturation (looking at the expression of MAP2 and βIII-tubulin) [42]. A neurogenesis-enhancing effect of laminin has also been reported previously, but when assessing the generation of neural progenitors starting from human embryonic stem cells [34]. Notably, another recent study revealed that laminin α2β1γ1 (laminin211) regulates the proliferation and differentiation of human embryonic stem cell-derived midbrain dopaminergic (mDA) neurons in a concentration-dependent manner, with high concentrations of lm211 promoting the progenitor identity and reducing TH+ differentiation [43]. Several studies have reported that laminins also promote the survival of oligodendrocyte lineage cells [44,45] and regulate oligodendrocyte differentiation [46,47]. Here, we did not detect any significant effect of laminin on the acquisition of the oligodendroglial cell fate of NSCs. Experimental work on extracellular matrix scaffolds and NSC differentiation has produced contradicting results on NSC-derived oligodendrogenesis, indicating the involvement of intricate and different mechanisms. One study reported that when NSCs were cultured in a methylcellulose scaffold functionalized with laminin, OPC generation was increased [48], while co-immobilizing laminin with several neurotrophic factors on a culture surface inhibited this process [49].

Because integrin-β1 has been extensively shown to regulate NSC behavior and maintenance in the brain [16,50], mainly via a laminin/integrin-β1 interaction [51], we chose to block its function. Integrins bearing a β1 subunit are expressed by all active neural stem and progenitor cells [16] and, in addition, control cell chain formation in the Rostral Migratory Stream [52], and loss of this heterodimer provokes a decrease in the proliferation of nestin-progenitor cells [53]. In our experiments, integrin-β1 blocking provoked a significant decrease in neurogenesis, almost eliminating the appearance of Dcx+ cells. Our results are consistent with a previous study in which ablation of β1-integrin induced a significant reduction in the number of Dcx+ neuroblasts in the dentate gyrus stem cell niche of the hippocampus [54]. Importantly, our data revealed that integrin-β1 does not mediate the effects of laminin as a repressor of progress towards the neuronal fate, as its blocking did not reverse this effect. However, it is noteworthy that the ‘β1′ subunit can form dimers with a range of ‘a’ subunits and therefore the inhibition of different β1 heterodimers may promote various effects [19]. In addition, laminins can also interact with a range of other receptors, such as syndecans [55], which are involved in the regulation of adult neurogenesis in the SVZ [56]. The divergence in the properties of neural progenitors once they acquire divergent cell fates was also evident in the pro-oligodendrogenic effect of the blocking of integrin-β1. Based on evidence that integrins-β1 are key receptors for the survival and differentiation of OPCs [46,57], this was an unexpected result. However, in the context of glial cells, it has been proposed that conditional ablation of b1-integrin increases the astrogliogenesis of SVZ progenitor cells [58], and that loss-of-function of dystroglycan, the second major laminin receptor which is expressed in oligodendrocyte lineage cells, induces an overproduction of OPCs in the SVZ and in cell cultures, with neurospheres from deficient mice overproducing OPCs, with oligodendrocyte differentiation and maturation being delayed after dystroglycan loss [59].

As described above, in the polymorphic NSC assays, stem and progenitor cells behave in ways that are very relevant to in vivo observations. To examine this further, we investigated the effects of BNN-20, a synthetic microneurotrophin previously shown to affect NSCs specifically in the parenchyma (in the substantia nigra) and not in the SEZ or the hippocampus [33]. Interestingly, BNN-20 administration led to a significant increase in both neurogenesis and oligodendrogenesis only in the 2D areas of our cell culture system.

## 5. Conclusions

Here, we report the creation and analysis of polymorphic NSC cultures, using cells isolated from the SEZ niche. Our data reveal that in these assays, progenitors of neuronal and oligodendroglial commitment exhibit divergent properties regarding their spatial distribution, the role of the extracellular matrix component laminin, and of integrins. Notably, the differential properties of these progenitors in the cultures reflect differences that have been described in vivo. Therefore, these assays constitute a valid tool for the investigation of key properties of the brain’s stem cell populations and for predicting the effects of potential, progenitor-targeting molecules.

## Figures and Tables

**Table 1:** Summary of differences and similarities between neurogenesis and oligodendrogenesis, as tested or observed in neurosphere polymorphic in vitro assays (after seven days of differentiation). Red highlights the differences and green the similarities.

Microenvironment Factors	Neurogenesis	Oligodendrogenesis
**1. Levels of cell fate commitment**		
	2.28% ± 0.34 Dcx+ cells	9.30% ± 2.20 Olig2+ cells
**2. Cytoarchitecture**		
**2a. 3D vs. 2D**	− Significantly higher in 2D areas. − More often peripheral to 3D areas	No dependence
**2b. Cell density restrictions**	Appears in wider range of cell densities (on average 192 ± 21 cells per opt. field)	− Appears in narrower range of cell densities (on average 81 ± 5 cells per opt. field) − Limited aggregation (in 30 μm radius)
**2c. Correlation to cell density**	Significantly Positive (r = 0.722) → proportional	No correlation (r = −0.396)
**3. Laminin (niche ECM: leads to predominance of 2D areas)**		
**3a. Dependence on laminin**	Significant reduction in 2D areas	No dependence
**3b. Cell density restrictions**	− Appears in wider range of cell densities (on average 164 ± 17 cells per opt. field)	− Appears in narrower range of cell densities (on average 77 ± 4 cells per opt. field)
**3c. Correlation to cell density**	Significantly Positive (r = 0.800) → proportional	No correlation (r = −0.274)
**4. Integrin-β1 (effects of antibody-mediated blocking)**		
	Significantly reduced (3D & 2D) irrespective of substrate	− Significantly enhanced on PDL (3D) and on laminin (3D & 2D) − Reversal of aggregation restrictions (in 30 μm radius)
**5. Microneurotrophin BNN-20 (BDNF-mimicking)**		
	Significantly increased in 2D areas	Significantly increased in 2D areas

## Data Availability

The raw data supporting the data presented in this study are readily available on request from the corresponding author.

## Supplementary Materials

The following are available online at https://www.mdpi.com/article/10.3390/cells11111743/s1, Supplementary Figure S1. Validation of the cell profile between 2D areas of the polymorphic NSC assay and those of typical, adherent monolayer NSC cultures. (A–E) Graphs showing the comparison of total cell density (A) and of the percentages of Sox2+ (B), Dcx+ (C), beta-III tubulin+ (D) and Olig2+ (E) cells between 2D areas in the polymorphic NSC assay and the typical NSC monolayers after 7d of differentiation (*p* > 0.05, using paired *t*-test; Error bars: SEM; *n* = 2 samples). (F–I) Representative images of 2D areas in the polymorphic assay (F, H) and in monolayer cultures (G, I) after immunostaining for the above-mentioned markers. The arrows indicate cells of neuronal cell fate (Dcx + BIII+ cells). (J–L) Representative images from polymorphic NSC assays after immunostaining for Dcx and βΙΙΙ (in J), Olig2 and CC1 (in K) and Olig2 and PDGFRa (in L). The arrowheads indicate a mature neuron (Dcx + BIII+, in J), a mature oligodendrocyte (Olig2 + CC1+, in K) and OPCs (Olig2 + PDGFRa+, in L). [Scale bars, F–I: 10 μm and J–L: 40 μm]. Supplementary Figure S2. Analysis of the effects of laminin and of integrin-β1 blocking on neurogenesis and oligodendrogenesis. (A) Graph showing the presence of Olig2+ cells (either Olig2+ only, or double Olig2+ and PCNA+) and of non-Olig2+ cells around random Olig2+ cells. Data are shown as percentages. Note the statistically significant increase in the presence of Olig2+ cells when comparing the “control” versus “control + Ab” and “laminin” versus “laminin + Ab” groups. (* *p* < 0.05, using *t*-test analysis, *n* = 3; error bars: SEM). (B and C) Graphs showing the number (in B) and total length of processes (in C) in all experimental conditions (*p* > 0.05, Statistical analysis was performed by repeated measures ANOVA, *n* = 2–3). (D, D’ and E) Representative images which show characteristic morphologies of Dcx+ cells in control conditions or after the addition of β1-blocking antibody. The arrowheads and the arrow indicate Dcx+ cell bodies, respectively. [scale bar: 30 μm] Supplementary Movie S1. Time lapse video of neurospheres cultured on PDL. 72 h time-lapse imaging (with photos taken every 60 min), of neurospheres plated on PDL-coated glass coverslips and cultured in differentiation conditions. Supplementary Movie S2. Time lapse video of neurospheres cultured on laminin. 72 h time-lapse imaging (with photos taken every 60 min), of neurospheres plated on [PDL + laminin]-coated glass coverslips and cultured in differentiation conditions.
