# Supplementary material for: Divergence between Neuronal and Oligodendroglial Cell Fate, in Postnatal Brain Neural Stem Cells, Leads to Divergent Properties in Polymorphic In Vitro Assays
Source: Cells. 2022 May 25;11(11):1743. doi: 10.3390/cells11111743 (PMC9179558; doi:10.3390/cells11111743)
Supplement: Supplementary file 1 [file cells-11-01743-s001.zip › cells-1630016-supplementary.pdf]

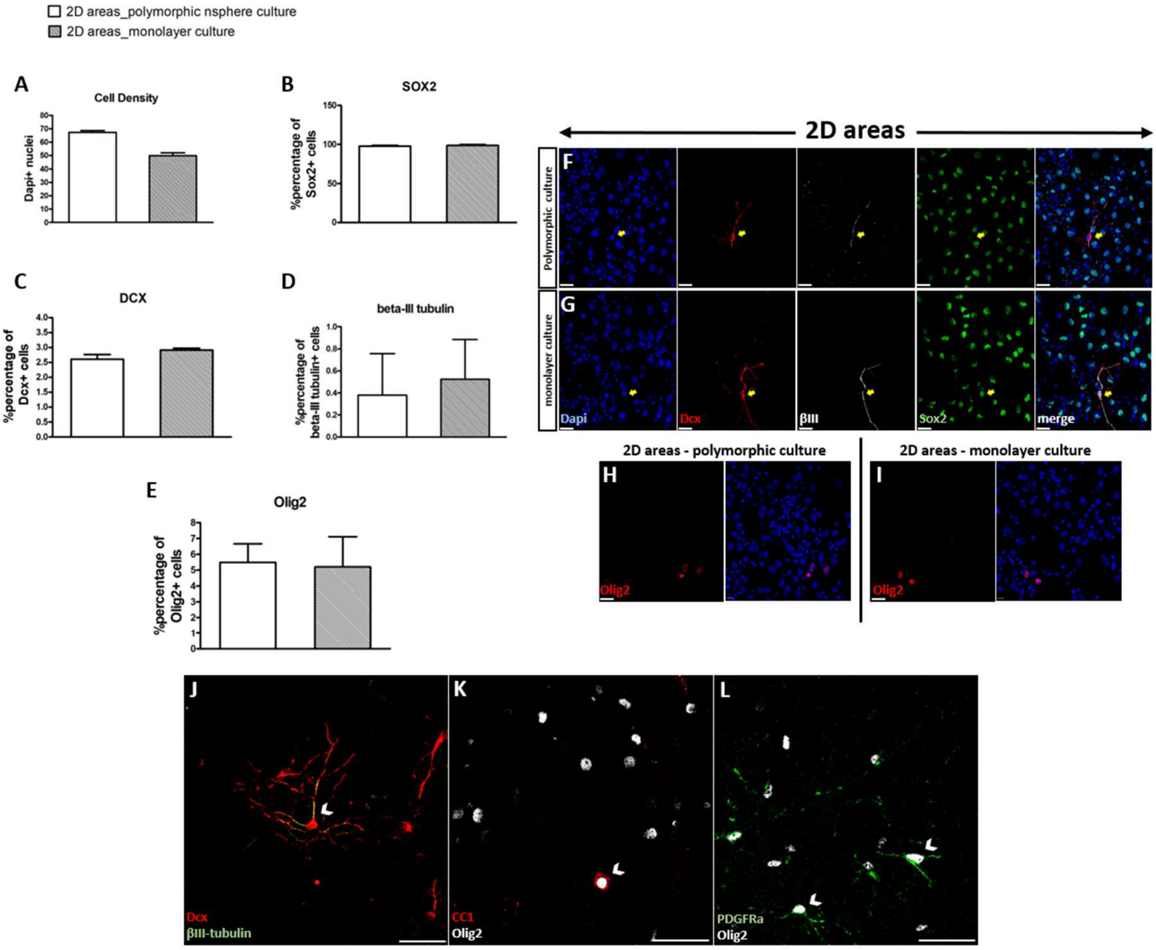

Figure S1. Validation of the cell profile between 2D areas of the polymorphic NSC assay and those of typical, adherent monolayer NSC cultures. (A-E) Graphs showing the comparison of total cell density (A) and of the percentages of Sox2+ (B), Dcx+ (C), beta-III tubulin+ (D) and Olig2+ (E) cells between 2D areas in the polymorphic NSC assay and the typical NSC monolayers after 7d of differentiation ($p > 0.05$, using paired t-test; Error bars: SEM; $n = 2$ samples). (F-I) Representative images of 2D areas in the polymorphic assay (F, H) and in monolayer cultures (G, I) after immunostaining for the above-mentioned markers. The arrows indicate cells of neuronal cell fate (Dcx + BIII+ cells). (J-L) Representative images from polymorphic NSC assays after immunostaining for Dcx and βIII (in J), Olig2 and CC1 (in K) and Olig2 and PDGFRa (in L). The arrowheads indicate a mature neuron (Dcx + BIII+, in J), a mature oligodendrocyte (Olig2 + CC1+, in K) and OPCs (Olig2 + PDGFRa+, in L). [Scale bars, F-I: 10 μm and J- L: 40μm].

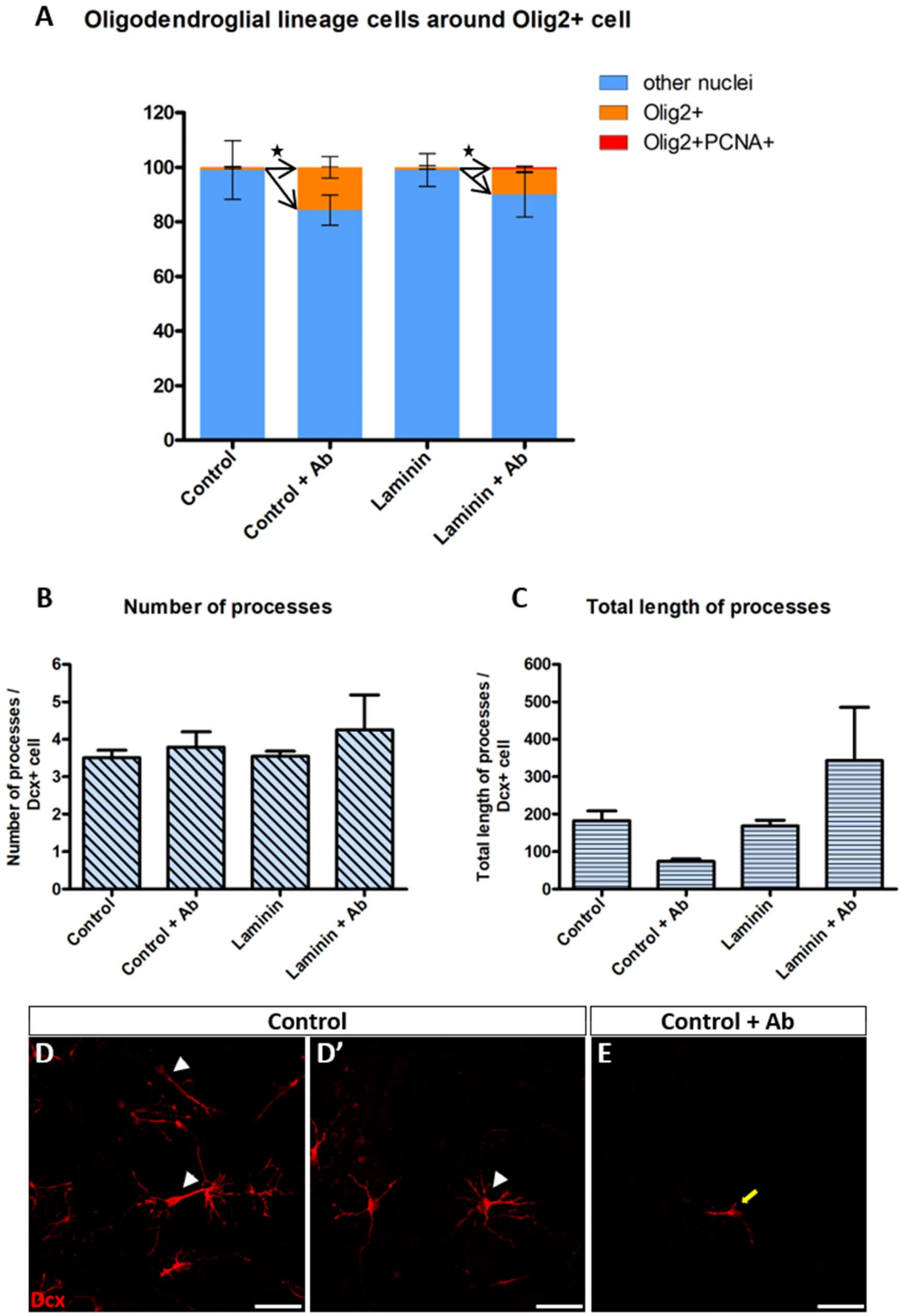

Supplementary Figure S2. Analysis of the effects of laminin and of integrin-β1 blocking on neurogenesis and oligodendrogenesis. (A) Graph showing the presence of Olig2+ cells (either Olig2+ only, or double Olig2+ and PCNA+) and of non-Olig2+ cells around random Olig2+ cells. Data are shown as percentages. Note the statistically significant increase in the presence of Olig2+ cells when

comparing the "control" versus "control + Ab" and "laminin" versus "laminin + Ab" groups. (*$p < 0.05$, using t-test analysis, $n = 3$; error bars: SEM). (B and C) Graphs showing the number (in B) and total length of processes (in C) in all experimental conditions ($p > 0.05$, Statistical analysis was performed by repeated measures ANOVA, $n = 2–3$). (D, D′ and E) Representative images which show characteristic morphologies of Dcx+ cells in control conditions or after the addition of β1-blocking antibody. The arrowheads and the arrow indicate Dcx+ cell bodies, respectively. [scale bar: 30μm]
